# Microbial and molecular differences according to the location of head and neck cancers

**DOI:** 10.1186/s12935-022-02554-6

**Published:** 2022-03-26

**Authors:** Yun Kyeong Kim, Eun Jung Kwon, Yeuni Yu, Jayoung Kim, Soo-Yeon Woo, Hee-Sun Choi, Munju Kwon, Keehoon Jung, Hyung-Sik Kim, Hae Ryoun Park, Dongjun Lee, Yun Hak Kim

**Affiliations:** 1 Convergence Medical Sciences, Pusan National University, Yangsan, Republic of Korea; 2 Interdisciplinary Program of Genomic Science, Pusan National University, Yangsan, Republic of Korea; 3 Biomedical Research Institute, Pusan National University Hospital, Busan, Republic of Korea; 4 Department of Convergence Medicine, School of Medicine, Pusan National University, 49 Busandaehak-ro, Yangsan, 50612 Republic of Korea; 5 Department of Anatomy and Cell Biology and Department of Biomedical Sciences, Seoul National University College of Medicine, Seoul, Republic of Korea; 6 Department of Oral Pathology, School of Dentistry, Pusan National University, Yangsan, Republic of Korea; 7 Periodontal Disease Signaling Network Research Center, School of Dentistry, Pusan National University, Yangsan, Republic of Korea; 8 Department of Anatomy, School of Medicine, Pusan National University, 49 Busandaehak-ro, Yangsan, 50612 Republic of Korea; 9 Department of Biomedical Informatics, School of Medicine, Pusan National University, Yangsan, Republic of Korea

**Keywords:** Microbiome, HNSCC, Oral cancer, Non-oral cancer, TCGA, KEGG pathway, Linear discriminant analysis

## Abstract

**Background:**

Microbiome has been shown to substantially contribute to some cancers. However, the diagnostic implications of microbiome in head and neck squamous cell carcinoma (HNSCC) remain unknown.

**Methods:**

To identify the molecular difference in the microbiome of oral and non-oral HNSCC, primary data was downloaded from the Kraken-TCGA dataset. The molecular differences in the microbiome of oral and non-oral HNSCC were identified using the linear discriminant analysis effect size method.

**Results:**

In the study, the common microbiomes in oral and non-oral cancers were *Fusobacterium, Leptotrichia, Selenomonas and Treponema* and *Clostridium and Pseudoalteromonas*, respectively. We found unique microbial signatures that positively correlated with Kyoto Encyclopedia of Genes and Genomes (KEGG) pathways in oral cancer and positively and negatively correlated KEGG pathways in non-oral cancer. In oral cancer, positively correlated genes were mostly found in prion diseases, Alzheimer disease, Parkinson disease, Salmonella infection, and Pathogenic Escherichia coli infection. In non-oral cancer, positively correlated genes showed Herpes simplex virus 1 infection and Spliceosome and negatively correlated genes showed results from PI3K-Akt signaling pathway, Focal adhesion, Regulation of actin cytoskeleton, ECM-receptor interaction and Dilated cardiomyopathy.

**Conclusions:**

These results could help in understanding the underlying biological mechanisms of the microbiome of oral and non-oral HNSCC. Microbiome-based oncology diagnostic tool warrants further exploration.

**Supplementary Information:**

The online version contains supplementary material available at 10.1186/s12935-022-02554-6.

## Introduction

Head and neck squamous cell carcinoma (HNSCC) is the sixth most common cancer worldwide, with 890,000 new cases and 450,000 deaths in 2018 [1, 2]. HNSCC accounts for about 6% of all cancers and 1–2% of deaths due to neoplastic diseases [3–5]. HNSCC is a heterogeneous disease and tumours are distinguished based on location. HNSCC originates from the epithelial cells in the laryngeal and oropharynx, lips, mouth or larynx. Tobacco and alcohol consumption are the well-known and geographically most prevalent risk factors for HNSCC [6]. Heavy users of these carcinogens-containing products have a 35-fold higher risk of developing HNSCC than non-users [6, 7], and approximately three-quarters of HNSCC cases attributable to cigarette smoking and tobacco use [8]. In addition, betel nut chewing is independent risk factor for HNSCC in India, China or Taiwan [9, 10]. Especially, development of oropharyngeal cancers is strongly associated with HPV infection, which mainly occurs in Western Europe and the United States [6, 11].

Trillions of microbes have evolved and continue to live on and within human beings [12]. Numerous studies have suggested a link between the microbiota, which exist in various organs (e.g., gut and placenta) and pathological conditions such as neurologic diseases, metabolic disorders, and cancers [13–16]. With the development of omics technologies, such as metagenomics, transcriptomics, and proteomics, substantial evidence has been accumulated regarding the relationship of microorganisms and various diseases, including cancers [17].

The gut microbiome has been associated with various disorders, especially malignant tumours. The gut microbiome is involved in biological processes, including modulating the metabolic phenotype, regulating epithelial development, and influencing innate immunity [18]. Chronic diseases such as obesity, inflammatory bowel disease, diabetes mellitus, metabolic syndrome, atherosclerosis, alcoholic liver disease, non-alcoholic fatty liver disease, cirrhosis are associated with the human microbiome [19]. Several studies have demonstrated that gut microbiome dysbiosis is associated with tumourigenesis and/or tumour growth across cancer types, including colon, hepatocellular carcinoma, gastric, and breast [13, 18]. Moreover, the gut microbiome has been demonstrated to play a key role in the response to cancer therapy, such as chemotherapy, immune checkpoint blockade, and stem cell transplant [13]. For immune checkpoint blockade response, differential gut microbiome signatures exist in patients who respond to immune checkpoint blockade treatment [20–22].

Although intratumoral microbiota has not been studied as much as the gut microbiota, the importance of microbiota in tumours is increasing, with studies showing that it affects the response to cancer treatment [13, 23–26]. Intratumoral bacteria, which are metabolically active, can alter the chemical structure of anti-cancer drugs [27, 28]. In addition, *Fusobacterium nucleatum* in colorectal tumour promotes resistance to chemotherapy through modulation of autophagy [29]. HNSCC, especially oral squamous cell carcinoma (OSCC), is the most prevalent and commonly studied cancer associated with bacterial infection, and is the most common malignancy of the head and neck worldwide [30]. Two prominent oral pathogens, *Porphyromonas gingivalis*, and *F. nucleatum* have been reported to promote tumour progression in mice [31]. Periodontitis is an infectious disease causing chronic inflammation in the oral cavity [32, 33]. Periodontitis has been linked to various cancers, including oesophageal and oropharyngeal cancers [30]. Several studies have found that the risk of developing OSCC may increase with periodontal disease [34, 35], and periodontal disease increases the risk of oral cancer even after adjusting for significant risk factors [36, 37]. Herein, we investigated the underlying molecular differences of the microbiome of oral cancer and non-oral HNSCC.

## Methods

### Microbiome datasets & TCGA RNA-sequencing datasets

We downloaded Kraken-TCGA(The Cancer Genome Atlas) -Raw-Data (n = 17,625) from microbial count datasets [38] for this study. Primary tumours were selected from HNSCC of microbiome data, classified into RNA and WGS, and combined with TCGA clinical information to separate oral and non-oral subtype. RNA-expression sequencing and clinical data sets of HNSCC samples were downloaded from the Broad GDAC Firehose [39] on 20 Feb 2020. The samples were categorised based on the site of occurrence as either oral cancer (alveolar ridge, buccal mucosa, floor of the mouth, hard palate, lip, oral cavity, and oral tongue) or non-oral cancer (base of tongue, hypopharyngeal, larynx, oropharynx, and tonsil) (Supplementary Table). Preprocessing was used with the R program (version 4.0.3) [40].

### Linear discriminant analysis effect size (LEfSe)

To identify significantly different bacteria (as biomarkers) between the two groups at the genus level, taxa summaries were reformatted and inputted into LEfSe via the Huttenhower Lab Galaxy Server [41]. The LDA values of oral and non-oral HNSCC microbiome data of RNA and DNA were obtained. We used the LDA method to estimate the effect size of the abundant genus level [41].

Then, we obtained common bacteria of RNA and DNA with the threshold on the logarithmic LDA score for discriminative features of 2.0108 (p < 0.0076). In the settings of LEfSe, the Kruskal–Wallis sum-rank test (α = 0.05) was used to detect taxa with significant differential abundance.

### Phylogenetic investigation of communities by reconstruction of unobserved states (PICRUSt) and ANOVA-like differential expression (ALDEx2)

The name of the common bacteria was changed to ID of Greengenes (97% taxonomy) (version 13.5) (http://greengenes.lbl.gov) and used as an input file. PICRUSt was performed using the Galaxy web application, which was used to predict bacterial metabolic contributions of oral rich and non-oral rich bacteria, respectively [42]. To filter the results of the PICRUSts, we merged results of oral rich and non-oral rich bacteria, and used the ALDEx2 [43] to obtain top five pathways with a p-value of 0.05 or less.

### Correlation analysis

A correlation analysis was performed with respect to the RNA expression data and common bacteria data of oral and non-oral HNSCC. Using the Spearman correlation test, genes with oral/non-oral correlation coefficients r > 0.15 and r < − 0.15 were obtained. Significance levels were considered at P < 0.05.

### Protein–protein interaction (PPI) analysis & Hub gene

PPI analysis of correlated genes was performed using the plug-in Search Tool for the Retrieval of Interacting Genes (STRING) app (version 1.5.1) [44]. The results of the analysis were imported into Cytoscape (version 3.8.2) [45] to establish a network model. The plug-in app cytohubba (version 0.1) [46] in Cytoscape was downloaded and installed. The top ten scores of the degree algorithm were taken as the criteria to screen out the hub genes with high connectivity in the gene expression network.

### KEGG pathway and gene ontology (GO)

KEGG pathway and GO analysis were performed on the DAVID website [47] with the genes in the node table resulting from the PPI. Then, the genetic symbol was transferred to entrezID using the org.Hs.eg.db (version 3.12.0) package [48] with the same input file from the PPI for subsequent analysis. The results of enhanced GO entries and KEGG were visualised as path point plots using clusterProfiler (version 3.18.1), ggplot (version 3.3.5), and Enrichplot2 (version 1.10.2) packages. GO and KEGG analysed the used data with statistically significant false discovery rates < 0.05.

## Results

### Characterisation of unique microbial signatures of oral and non-oral HNSCC

**Fig. 1:**
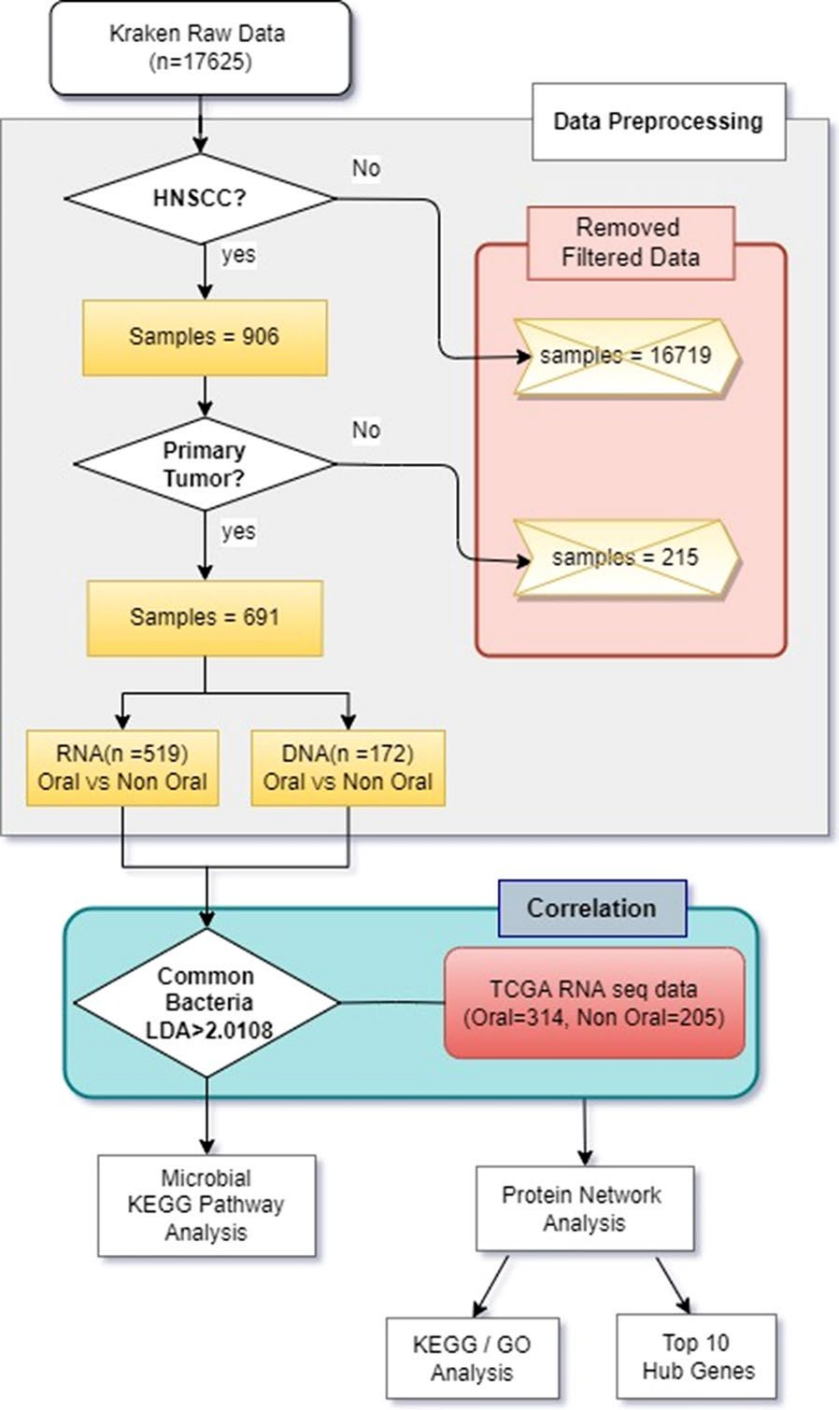
Pipeline flow chart throughout the study

To evaluate the unique microbial signatures of oral and non-oral HNSCC, we analysed Kraken-TCGA data sets using the linear discriminant analysis (LDA) method. We divided 691 HNSCC samples into 172 DNA whole genome sequencing (WGS) data and 519 RNA sequencing data (Fig. 1). Next, we analysed RNA sequencing as subtypes divided into 314 oral cancer and 205 non-oral cancer. DNA WGS data were also analysed as 115 oral and 57 non-oral subtypes. Clinical information related to these samples is described in Table 1. In both data, gender (P = 8.698E-05 (RNA)/2.372E-06 (DNA)) HPV status (P = 1.623E-09 (RNA)/5.201E-08 (DNA)), clinical stage (P = 3.998E-03 (RNA)/1.100E-03 (DNA)) and pathologic stage (P = 4.998E-04 (RNA)/2733E-05 (DNA)) were significantly different between patients with oral and non-oral cancers.

**Table 1:** Patient’s characteristics

Variables		RNA (*N* = 519)					Variables		DNA (*N* = 172)				
		Oral (314)		Non-oral (205)		P-value			Oral (115)		Non-oral (57)		P-value
Age	< 66	202	(64%)	154	(75%)	0.030*	Age	< 66	80	(70%)	43	(75%)	0.476
	≥ 66	111	(35%)	51	(25%)			≥ 66	35	(30%)	14	(25%)	
	NA	1	(0%)	–	–			NA	–	–	–	–	
Gender	Female	102	(32%)	34	(17%)	8.698E-05***	Gender	Female	41	(36%)	–	–	2.372E-06***
	Male	212	(68%)	171	(83%)			Male	74	(64%)	51	(89%)	
HPV status	positive	32	(10%)	65	(32%)	1.623E-09***	HPV status	Positive	16	(14%)	31	(54%)	5.201E-08***
	negative	282	(90%)	140	(68%)			Negative	99	(86%)	26	(46%)	
	NA	1	(0%)	–	–			NA	–	–	–	–	
Clinical Stage	Stage I	12	(4%)	8	(4%)	3.998E-03** 4.998E-04***	Clinical stage	Stage I	4	(3%)	–	–	1.100E-03**
	Stage II	76	(24%)	22	(11%)			Stage II	29	(25%)	9	(16%)	
	Stage III	65	(21%)	40	(20%)			Stage III	29	(25%)	7	(12%)	
	Stage IVA	146	(46%)	118	(58%)			Stage IVA	53	(46%)	35	(61%)	
	Stage IVB	4	(1%)	7	(3%)			Stage IVB	–	–	4	(7%)	
	Stage IVC	3	(1%)	4	(2%)			Stage IVC	–	–	1	(2%)	
	NA	8	(3%)	6	(3%)			NA	–	–	1	(2%)	
Pathologic Stage	StageI	21	(7%)	6	(3%)		Pathologic stage	Stage I	9	(8%)	2	(4%)	2.733E-05***
	Stage II	54	(17%)	20	(10%)			Stage II	22	(19%)	4	(7%)	
	Stage III	56	(18%)	25	(12%)			Stage III	18	(16%)	8	(14%)	
	Stage IVA	154	(49%)	98	(48%)			Stage IVA	56	(49%)	19	(33%)	
	Stage IVB	7	(2%)	5	(2%)			Stage IVB	1	(1%)	2	(4%)	
	Stage IVC	–	–	1	(0%)			Stage IVC	–	–	–	–	
	NA	22	(7%)	50	(24%)			NA	9	(8%)	22	(39%)	
Race	American Indian or Alaska native	1	(0%)	1	(0%)	0.029*	Race	American Indian or Alaska native	–	–	–	–	0.379
	Asian	10	(3%)	1	(0%)			Asian	2	(2%)	–	–	
	Black or African American	22	(7%)	26	(13%)			Black or African American	6	(5%)	6	(11%)	
	White	270	(86%)	173	(84%)			White	105	(91%)	51	(89%)	
	NA	11	(4%)	4	(2%)			NA	2	(2%)	–	–	
Alcohol History	Yes	202	(64%)	144	(70%)	0.393	Alcohol history	Yes	72	(63%)	48	(84%)	1.913E-03**
	NO	105	(33%)	57	(28%)			NO	41	(36%)	7	(12%)	
	NA	7	(2%)	4	(2%)			NA	2	(2%)	2	(4%)	
Pack Years Smoked	30 <	52	(17%)	37	(18%)	0.014*	Pack years smoked	30 <	16	(14%)	12	(21%)	0.174
	30≥	111	(35%)	95	(46%)			30 ≥	42	(37%)	25	(44%)	
	NA	151	(48%)	73	(36%)			NA	57	(50%)	20	(35%)	

AJCC version:4–7th, P < 0.05 ** P < 0.01 ***P < 0.001, HNSCC, head and neck squamous cell carcinoma; *NA* not available

Chi-squared test was done for gender, HPV status, Pack Years Smoked and Fisher’s exact-test was done for Age, Clinical Stage, Pathologic Stage, Race, Alcohol History

### Investigation of the common microbiome of oral and non-oral HNSCC

**Fig. 2:**
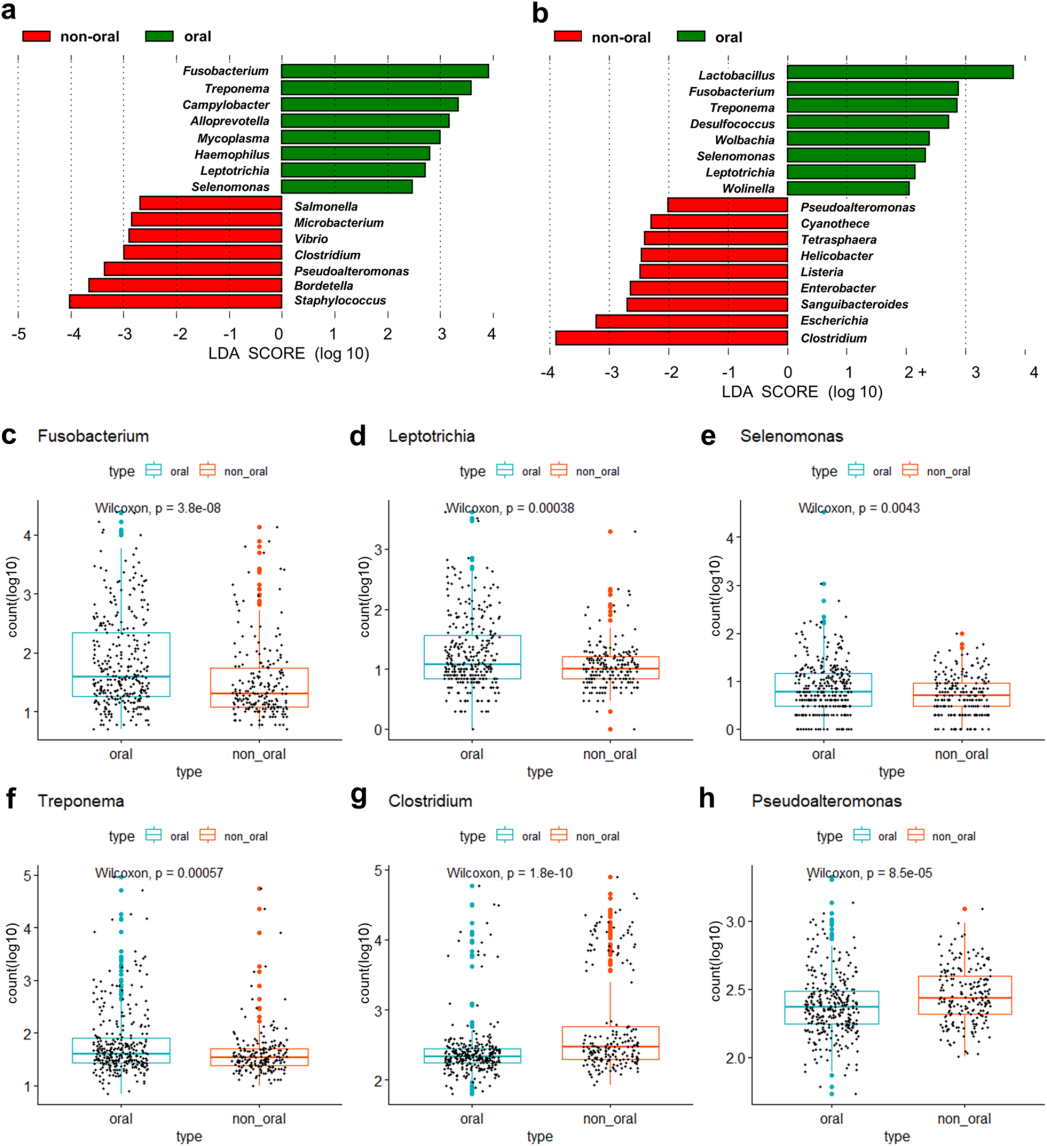
Linear discriminant analysis effect size (LEfSe) analyses and distribution of the microbiome by subtype. LEfSe analysis of microbiome composition between oral and non-oral-associated cancers was performed on **a** bacterial DNA and **b** bacterial RNA, respectively. Bacteria species enriched in oral cancer had a positive linear discriminant analysis (LDA) score, while bacteria species enriched in non-oral cancer had a negative score. Microbiomes with higher levels of distribution in oral cancer were **c** *Fusobacterium*, **d** *Leptotrichia*, **e** *Selenomonas, and* **f** *Treponema.* Microbiomes with higher levels of distribution in non-oral cancer were **f** *Clostridium* and **g** *Pseudoalteromonas*

The relatively enriched microbiome of oral and non-oral HNSCC are shown in Fig. 2a, b. The enriched microbiomes in oral HNSCC were *Fusobacterium, Leptotrichia, Selenomonas* and *Treponema* and the enriched microbiomes in non-oral HNSCC were *Clostridium* and *Pseudoalteromonas*, as determined by the linear discriminant analysis effect size (LEfSe) method (Fig. 2a, b). The distribution of count data for each microbiome subtypes is depicted in Fig. 2c–h.

### Microbial Kyoto Encyclopedia of Genes and Genomes (KEGG) pathway and protein network of oral and non-oral HNSCC

**Fig. 3:**
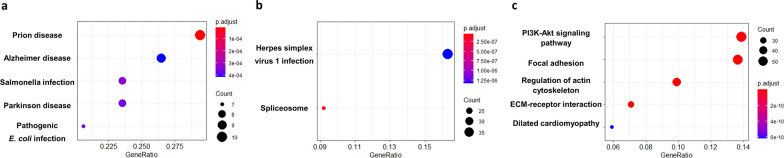
Kyoto Encyclopedia of Genes and Genomes (KEGG) pathway enrichment analysis. **a** Significantly enriched KEGG pathways of the positively correlated genes in oral cancer. **b** Significantly enriched KEGG pathways of the positively correlated genes in non-oral cancer. **c** Significantly enriched KEGG pathways of the negatively correlated genes in non-oral cancer. The left Y-axis shows the KEGG pathway. The X-axis shows the gene ratio

**Fig. 4:**
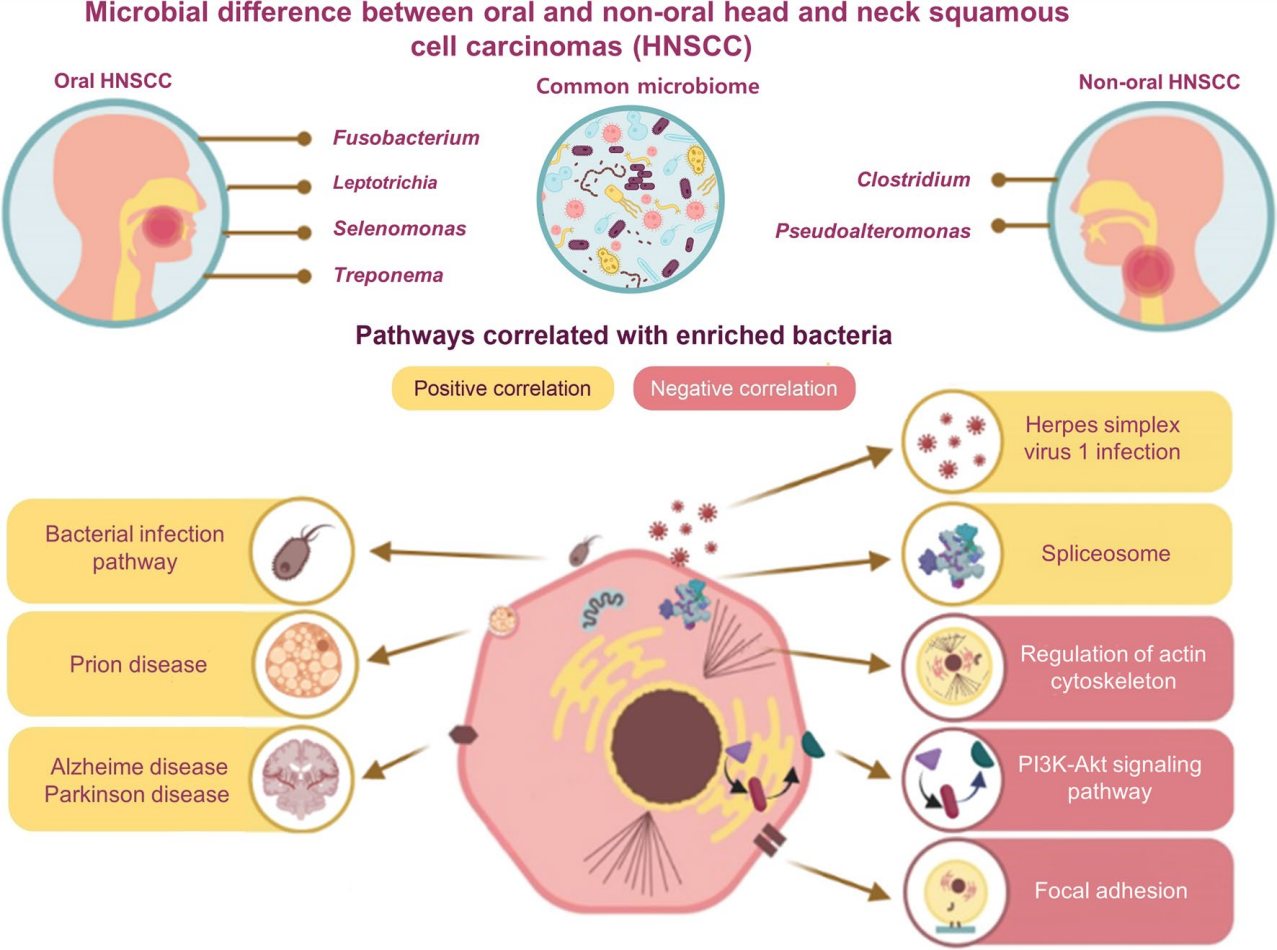
Graphical summary of this study

We analysed the molecular mechanism of the microbiome of oral and non-oral HNSCC using KEGG pathway analysis and protein network analysis (Fig. 3, Tables 1 and 2). We found unique microbial signatures that positively correlated KEGG pathways in oral HNSCC, positively correlated KEGG pathways and negatively correlated KEGG pathways in non-oral HNSCC (Figs. 3 and 4). In oral HNSCC, positively correlated genes were mostly found in bacterial infection pathways, and the genes involved in neurodegenerative diseases (prion diseases, Alzheimer disease, and Parkinson disease). In non-oral cancer, positively correlated genes were found Herpes simplex virus 1 infection and Spliceosome and negatively correlated genes showed results from PI3K-Akt signaling pathway, focal adhesion and regulation of actin cytoskeleton and Dilated cardiomyopathy. In addition, we conducted a pathway and gene expression analysis using microbial data of subtypes from each oral and non-oral HNSCC. As a result of PICRUSt, rich microbiome within oral cancer was involved in germination, Huntington's disease, biosynthesis of siderophore group nonribosomal peptides, atrazine degradation and prion diseases. Rich microbiome within non-oral cancer was found to be associated with other glycan degradation, Lysosome, Glycosphingolipid biosynthesis—globo series, electron transfer carriers, and glycosaminoglycan degradation (Table 2 and Additional file 2: Table S1). Rich microbiome within non-oral cancer was found to be associated with biosynthesis and metabolism of glycan, transport, catabolism, and biosynthesis of other secondary metabolites. Rich microbiome within oral cancer was involved in the biodegradation and metabolism of xenobiotics, neurodegenerative diseases, and the circulatory system. We found significant pathways using correlated genes with microbiome. We identified the KEGG pathways by selecting only the nodded genes as a protein–protein interaction tool (Table 3). The results of the phylogenetic investigation of communities by reconstruction of unobserved states (PICRUSt) analysis are shown in Additional file 1: Fig. S1. ALDEx2 was performed by merging the KEGG pathways obtained after PICRUSt of each subtype. The result is the median expression value of the KEGG pathway, and is expressed as a dot on the graph (Additional file 2: Table S1).

**Table 2:** Results of PICRUSt KEGG pathway enrichment analysis

	Level 1	Level 2	Level 3	Rab.win. non-oral	Rab.win.oral	diff.btw
Oral rich bacteria	Unclassified	Cellular Processes and Signaling	Germination	− 0.421	8.339	9.005
	Human diseases	Neurodegenerative diseases	Huntington's disease	0.541	8.378	7.972
	Metabolism	Metabolism of Terpenoids and Polyketides	Biosynthesis of siderophore group nonribosomal peptides	1.232	8.327	7.111
	Metabolism	Xenobiotics Biodegradation and Metabolism	Atrazine degradation	1.559	8.333	6.271
	Human diseases	Neurodegenerative diseases	Prion diseases	2.353	8.343	6.171
Non-oral rich bacteria	Metabolism	Glycan biosynthesis and metabolism	Other glycan degradation	10.295	− 0.612	− 10.855
	Cellular processes	Transport and catabolism	Lysosome	10.270	− 0.458	− 10.537
	Metabolism	Glycan biosynthesis and metabolism	Glycosphingolipid biosynthesis—globo series	10.230	0.474	− 9.590
	Unclassified	Cellular processes and signaling	Electron transfer carriers	10.290	1.406	− 8.713
	Metabolism	Glycan biosynthesis and metabolism	Glycosaminoglycan degradation	10.275	1.553	− 8.588

BH < 0.05 compared to the oral and non-oral (ALDEx2); *BH* Benjamini-Hochberg

diff.btw cut off > abs(6)

rab.win.non-oral: a vector containing the median clr value for each feature in non-oral, *clr* centred log-ratio

rab.win.oral: a vector containing the median clr value for each feature in oral

diff.btw: a vector containing the per-feature median difference between condition non-oral and oral

*PICRUSt* phylogenetic investigation of communities by reconstruction of unobserved states; *KEGG* Kyoto Encyclopedia of Genes and Genomes

**Table 3:** DAVID gene-annotation enrichment analysis of KEGG pathway

	ID	KEGG pathway	Count	P-value	FDR	Genes
Positively correlated genes in oral cancer	hsa05020	Prion disease	10	9.21E-07	9.120E-05	*STIP1, PSMA6, TUBA1C, PSMD12, TUBB6, TUBB2A, IL1B, PPIF, TUBB4B, TUBA4A*
	hsa05010	Alzheimer disease	9	1.15E-04	2.412E-03	*PSMA6, TUBA1C, PSMD12, TUBB6, TUBB2A, IL1B, PPIF, TUBB4B, TUBA4A*
	hsa05132	Salmonella infection	8	5.09E-05	2.412E-03	*TUBA1C, TUBB6, TUBB2A, CXCL8, IL1B, TUBB4B, DYNLL1, TUBA4A*
	hsa05012	Parkinson disease	8	7.74E-05	2.412E-03	*PSMA6, TUBA1C, PSMD12, TUBB6, TUBB2A, PPIF, TUBB4B, TUBA4A*
	hsa05130	Pathogenic Escherichia coli infection	7	1.22E-04	2.412E-03	*TUBA1C, TUBB6, TUBB2A, CXCL8, IL1B, TUBB4B, TUBA4A*
Positively correlated genes in oral cancer	hsa05168	Herpes simplex virus 1 infection	39	6.31067961	3.342E-08	*ZNF155, ZNF132, ZNF550, ZNF195, ZNF606, ZNF84, ZNF823, ZNF547, ZNF205, ZNF766, ZNF600, ZNF226, ZNF302, EIF2B1, ZNF566, ZNF620, ZNF224, ZNF564, ZNF443, ZNF584, ZNF441, ZNF141, ZNF140, ZNF283, BST2, IRF3, ZNF519, IRF7, SRSF2, SRSF3, ZNF337, ZNF557, SRSF5, ZNF780A, SRSF6, SRSF7, ZNF112, ZNF530, ZNF354B*
	hsa03040	Spliceosome	22	3.55987055	1.454E-09	*PRPF38B, HSPA1L, RBM8A, CCDC12, THOC1, MAGOHB, LSM5, LSM4, LSM2, XAB2, HNRNPM, PHF5A, PRPF18, TRA2B, MAGOH, SRSF2, SRSF3, PRPF31, SRSF5, SRSF6, SRSF7, SRSF10*
Negatively correlated genes in non-oral cancer	hsa04151	PI3K-Akt signaling pathway	59	6.5701559	1.870E-15	*ITGB1, ATF2, FLT1, ITGB5, IRS1, ITGB4, FLT4, ITGB3, TNC, LAMC2, LAMC1, IGF1R, RPTOR, GYS1, PPP2R5E, CREB3L2, KDR, ITGAV, ITGB6, IL6R, YWHAG, PDGFRB, MAP2K1, ITGA3, ITGA1, F2R, PRKCA, OSMR, COL4A2, PIK3CA, COL4A1, COL6A1, COL6A3, ITGA7, ITGA6, ITGA5, ITGA9, CREB5, LAMA2, LAMA4, LAMA3, PDGFB, LPAR3, LPAR4, THBS2, THBS1, EGFR, RELA, RXRA, PDGFC, MAPK1, ANGPT2, LAMB3, FN1, PPP2R3A, COL1A1, COL1A2, ITGA11, TEK*
	hsa04510	Focal adhesion	58	6.45879733	1.455E-27	*ITGB1, FLT1, ITGB5, ITGB4, FLT4, ITGB3, TNC, LAMC2, LAMC1, ACTB, IGF1R, MYLK, KDR, ITGAV, ITGB6, PDGFRB, MAP2K1, ITGA3, ACTN1, ITGA1, PRKCA, ACTN4, COL4A2, PIK3CA, COL4A1, COL6A1, RAPGEF1, COL6A3, ITGA7, ITGA6, ITGA5, TLN1, CRK, VCL, ITGA9, LAMA2, ROCK2, LAMA4, PXN, LAMA3, PDGFB, THBS2, THBS1, EGFR, PDGFC, FLNA, MAPK1, FLNB, FLNC, PAK2, LAMB3, CAV1, FN1, PARVA, COL1A1, COL1A2, ITGA11, ZYX*
	hsa04810	Regulation of actin cytoskeleton	42	4.67706013	3.317E-13	*ITGB1, CYFIP1, ITGB5, ROCK2, ITGB4, ITGB3, ARPC1B, PXN, PDGFB, WASL, LPAR4, IQGAP1, EGFR, ACTB, SLC9A1, MYLK, GNA12, PDGFC, MAPK1, ITGAV, ITGB6, PAK2, PDGFRB, MAP2K1, ITGA3, ACTN1, LIMK1, ITGA1, F2R, FN1, MSN, ACTN4, ENAH, PIK3CA, ITGA11, MYH9, ITGA7, ITGA6, ITGA5, CRK, VCL, ITGA9*
	hsa04512	ECM-receptor interaction	30	3.34075724	1.945E-16	*ITGB1, LAMA2, ITGB5, ITGB4, LAMA4, ITGB3, LAMA3, TNC, LAMC2, LAMC1, THBS2, THBS1, ITGAV, ITGB6, LAMB3, ITGA3, ITGA1, FN1, HSPG2, COL1A1, COL1A2, COL4A2, COL4A1, COL6A1, ITGA11, COL6A3, ITGA7, ITGA6, ITGA5, ITGA9*
	hsa05414	Dilated cardiomyopathy	25	2.78396437	6.870E-11	*ITGB1, LAMA2, ITGB5, ITGB4, ITGB3, ATP2A2, ADCY1, ADCY7, ACTB, SGCD, SGCA, ITGAV, ITGB6, TPM4, ITGA3, TPM1, ITGA1, ACTC1, DES, ITGA11, MYL3, ITGA7, ITGA6, ITGA5, ITGA9*

*FDR* false discovery rate

## Discussion

The microbiome plays an important role in the human host and participates in the development of a wide variety of diseases, such as cancer [12]. The tumor microbiome is associated with a chronic inflammatory state and modulates the initiation and development of various cancers, such as lung, breast, colon, gastric, pancreatic, cholangiocarcinoma, ovarian, and prostate cancers [13, 23–26, 49–51]. In colorectal cancer (CRC), transplant of stool containing the tumor microbiome from patients with CRC can induce polyp formation [52, 53]. Moreover, some bacterial species (*F. nucleatum)* can stimulate an inflammatory state that can promote carcinogenesis via increased production of reactive oxygen species [54], induction of proinflammatory toxins [55, 56], and suppression of anti-tumor immune functions [57, 58]. In this study, for the first time, we differentiated the microbiota of HNSCC into oral and non-oral cancers to identify differences in the abundance of the tumor microbiome. Then, we then attempted a molecular approach using the correlation between the microbiome and mRNA expression. We systematically selected six microbiomes as unique microbial signatures of oral and non-oral HNSCC. Microbiomes with higher levels of distribution in oral HNSCC were *Selenomonas*, *Fusobacterium*, *Leptotrichia* and *Treponema,* while microbiomes with higher levels of distribution in non-oral HNSC were *Clostridium* and *Pseudoalteromonas*.

The relationship between oral microbiota and human diseases has studied a lot. Especially, several bacteria including *Porphyromonas gingivalis, Treponema denticola, Selenomonas sputigena* and *Fusobacterium nucleatum* have been associated with cancer development [59–61]. In the current study, we observed the *Fusobacterium, Treponema, Leptotrichia* were enriched in oral cancer compared to non-oral cancer. In consistent with previous research, it may have a negative effect on cancer progression. *Clostridium* species, which are well-studied anaerobic bacterium, has high ability for colonization in the hypoxic and necrotic lesions in tumour [62]. Genetically modified *Clostridium* expressing tumour suppressive genes is one of the therapeutic strategies of cancers. Since the *Clostridium* is enriched in non-oral cancer, it may be used as therapeutic options for non-oral cancers.

The prevention and treatment of diseases by targeting the microbiome have been widely investigated [30]. Modulation of the microbiome may also contribute to the treatment of cancer [63]. Cancer therapy requires an intact commensal microbiome that mediates the therapy effects by modulating functions of myeloid-derived suppressor cells in the tumor microenvironment [24, 63, 64]. Some studies have shown the deleterious effects of antibiotics on the treatment of cancer [13, 65]. Patients with metastatic renal cell carcinoma or non-small-cell lung cancer had significantly worse survival outcomes if they received antibiotics just before or just after the initiation of treatment with immune checkpoint blockade [66]. In addition, patients who received anti-Gram-positive antibiotics along with cyclophosphamide for chronic lymphocytic leukemia or cisplatin for relapsed lymphoma had a lower overall response rate [55, 67]. These microbiomes may confer susceptibility to certain cancers, either through a direct effect by the local presence within the tumor microenvironment or via the systemic impact of the microbiome from a distant location, such as the gut and the skin [68].

There are several limitations in this study. The results were not validated in other cohorts or experimental procedures. We obtained the results by using Kraken pipeline, which obtains microbiome information from whole genome sequencing or RNA sequencing data. Therefore, it is necessary to verify it by microbiome sequencing and/or PCR analysis.

Taken together, stress conditions, such as diet, antigen exposure, medications, and stress are important factors that contributing to the state of health and also affect the microbiome [38]. This field is young, and we are left with many unanswered questions—especially regarding the mechanism of action as well as the group of bacterial species that are most important in mediating antitumor effects. Multifaceted strategies are needed to modulate precision medicine and treat disease. Efforts are currently underway to enhance therapeutic responses and/or abrogate treatment-associated toxicity chemotherapeutic agents via modulation of the microbiome.

## Supplementary Information

**Additional file 1: Figure S1. **Output from ALDEx2 plot.

**Additional file 2: Table S1.** The GO analysis results, hub genes, and tumour locations of included patients.

## Data Availability

All the data were available on the manuscript or from the corresponding author on reasonable request.

## Abbreviations

TCGA: The Cancer Genome Atlas
ALDEx2: ANOVA-like differential expression tool for high-throughput sequencing data
CRC: Colorectal cancer
GO: Gene Ontology
HNSCC: Head and neck squamous cell carcinoma
KEGG: Kyoto Encyclopedia of Genes and Genomes
LDA: Linear discriminant analysis
LEfSe: Linear discriminant analysis effect size
OSCC: Oral squamous cell carcinoma
PICRUSt: Phylogenetic investigation of communities by reconstruction of unobserved states
PPI: Protein–protein interaction
STRING: Search Tool for the Retrieval of Interacting Genes/Proteins
TCGA: The Cancer Genome Atlas
WGS: Whole Genome equencing
