# Supplementary figures and images for: Microbial and molecular differences according to the location of head and neck cancers
Source: Cancer Cell Int. 2022 Mar 26;22:135. doi: 10.1186/s12935-022-02554-6 (PMC8962034; doi:10.1186/s12935-022-02554-6)

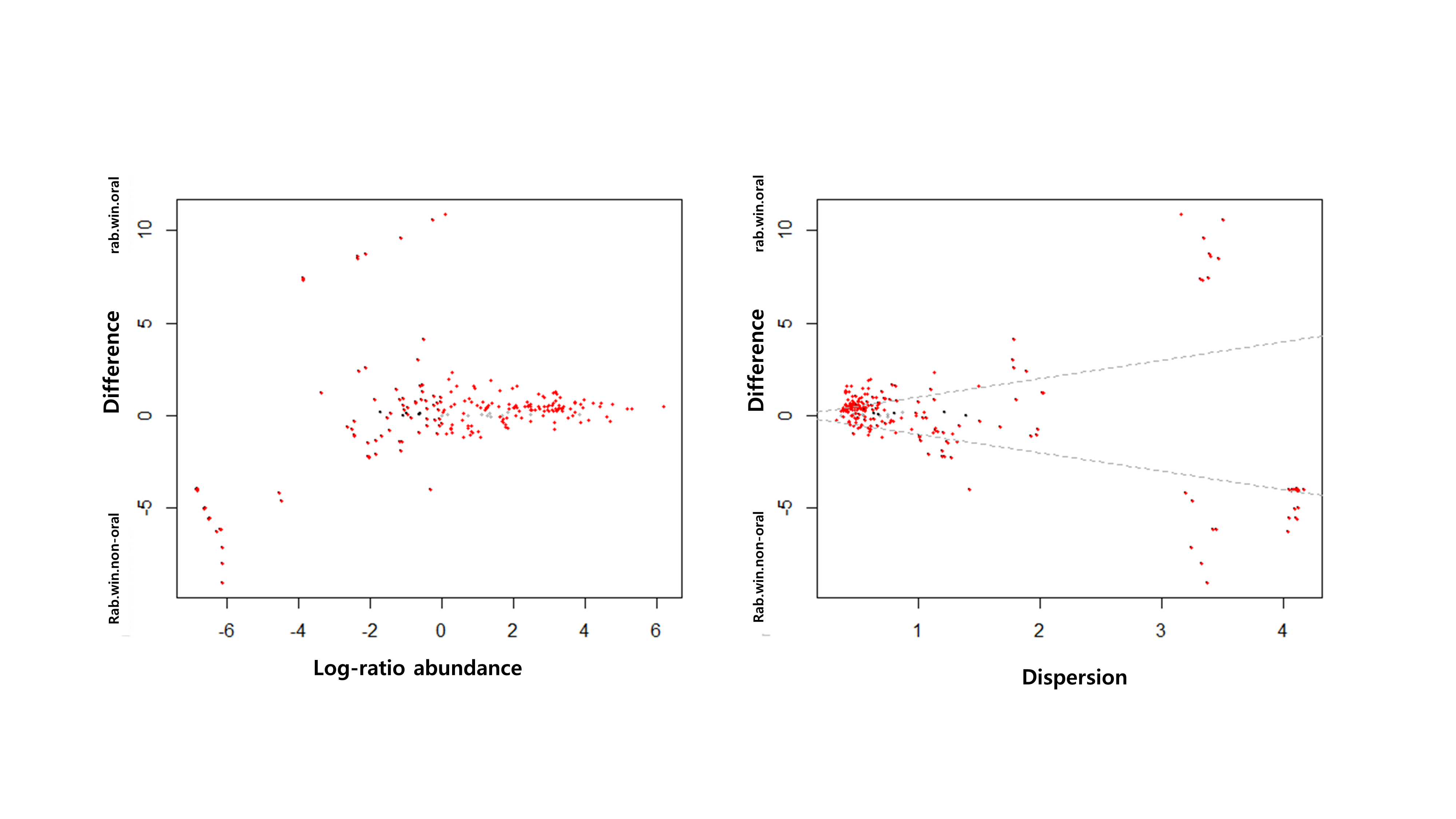

Supplement: Supplementary file 1 — Additional file 1: Figure S1. Output from ALDEx2 plot. [file 12935_2022_2554_MOESM1_ESM.tif]
